# Whole-Genome Sequencing and Biosynthetic Gene Cluster Analysis of Novel Entomopathogenic Bacteria *Xenorhabdus thailandensis* ALN 7.1 and ALN 11.5

**DOI:** 10.3390/biology14080905

**Published:** 2025-07-22

**Authors:** Wipanee Meesil, Jiranun Ardpairin, Liam K. R. Sharkey, Sacha J. Pidot, Apichat Vitta, Aunchalee Thanwisai

**Affiliations:** 1 Department of Microbiology and Parasitology, Faculty of Medical Science, Naresuan University, Phitsanulok 65000, Thailand; wipaneem61@nu.ac.th (W.M.); jiranuna61@nu.ac.th (J.A.); apichatv@nu.ac.th (A.V.); 2 Department of Microbiology and Immunology, Doherty Institute, 792 Elizabeth Street, Melbourne 3000, Australia; liam.sharkey@unimelb.edu.au (L.K.R.S.); sacha.pidot@unimelb.edu.au (S.J.P.); 3 Centre of Excellence in Medical Biotechnology (CEMB), Faculty of Medical Science, Naresuan University, Phitsanulok 65000, Thailand; 4 Center of Excellence for Biodiversity, Faculty of Sciences, Naresuan University, Phitsanulok 65000, Thailand

**Keywords:** biosynthetic gene clusters (BGCs), illumina sequencing, nanopore sequencing, natural products, symbiotic bacteria

## Abstract

*Xenorhabdus* species are entomopathogenic bacteria that live in symbiosis with *Steinernema* nematodes and produce a wide range of bioactive secondary metabolites. This study aimed to characterize the complete genomes and biosynthetic potential of two novel *Xenorhabdus* isolates, ALN7.1 and ALN11.5, recovered from *Steinernema lamjungense* collected in Northern Thailand. High-quality genome assemblies were generated, and phylogenomic comparisons confirmed that both isolates belonged to the recently described species *Xenorhabdus thailandensis*. The assembled genomes were approximately 4.02 Mb in size, each comprising a single circular chromosome with a GC content of 44.6% and encoding ~3800 protein-coding sequences, consistent with the features observed in other members of the genus. Biosynthetic gene cluster (BGCs) prediction using antiSMASH identified 19 BGCs in ALN7.1 and 18 in ALN11.5, including known clusters for holomycin, pyrrolizixenamide, hydrogen cyanide, and gamexpeptide C, along with several uncharacterized clusters, suggesting unexplored metabolic potential. Comparative analyses highlighted conserved yet strain-specific BGC profiles, indicating possible diversification within the species. These results provide genomic insights into *X. thailandensis* ALN7.1 and ALN11.5 and support their potential as valuable sources for the discovery of novel natural products and for future biotechnological applications.

## Simple Summary

Bacteria in the *Xenorhabdus* group live in partnership with tiny roundworms called *Steinernema*, which help them infect and kill insect pests. These bacteria are well known for producing many useful natural chemicals, some of which can be converted into new medicines or environmentally friendly pesticides. In this study, we examined two newly identified *Xenorhabdus* bacteria collected from *Steinernema* roundworms in Northern Thailand. Based on whole-genome sequencing, we confirmed that both strains belonged to *Xenorhabdus thailandensis*. We discovered that each of these bacteria has genetic instructions to produce many natural substances, including some that are already known to fight harmful germs and others that have not been studied before. Comparing the two bacteria showed that they share many important features but also have unique parts that may help them produce different, useful compounds. This research provides a starting point for using these bacteria to identify novel natural products that could benefit agriculture, medicine, and the environment.

## 1. Introduction

Bacterial secondary metabolites (also known as natural products) are non-essential compounds produced primarily during the stationary phase in response to nutrient limitation or environmental stress. These metabolites play key roles in ecological adaptation, defense, and interactions with the surrounding environment. Examples of secondary metabolites include phenazines, polyketides, nonribosomal peptides, ribosomal peptides, glucosides, alkaloids, and terpenoids [1]. They play an important role in therapeutic applications, such as antimicrobial, anticancer, anti-inflammatory, antioxidant, and immunosuppressive agents [2], and have potential applications in medicine and other industries [3,4]. Recent advances in genome-based approaches have revolutionized natural product discovery by enabling the efficient identification of bioactive compounds through the analysis of biosynthetic gene clusters (BGCs). These strategies facilitate early strain prioritization, allowing researchers to target microbial candidates with high biosynthetic potential and thereby reduce reliance on random screening methods [5,6]. Genome mining can also reveal cryptic or silent BGCs that remain inactive under standard laboratory conditions, uncovering previously hidden metabolic pathways and novel compound classes [6,7,8]. This genomics-driven approach has accelerated natural product discovery rates by up to 400% compared to traditional techniques [6], especially when integrated with synthetic biology, metabolomics, and automated screening platforms.

Entomopathogenic bacteria of the genus *Xenorhabdus* live in obligate symbiosis with nematodes of the genus *Steinernema* and play a critical role in the infection and death of insect hosts [9,10]. Beyond their ecological significance, *Xenorhabdus* species are prolific producers of diverse secondary metabolites, including antibiotics, cytotoxins, and virulence factors, many of which are encoded by biosynthetic gene clusters (BGCs) [11,12,13]. Several compounds derived from *Xenorhabdus* have demonstrated strong bioactivities and are being explored for their biotechnological applications. For example, odilorhabdin (ODL) is the most advanced antibiotic compound discovered in *Xenorhabdus* to date and has entered early clinical development [14,15]. Xenocoumacin and xenorhabdin exhibit potent antibacterial activity and have been proposed as leads for antibiotic development [16,17]. Other molecules, such as GameXPeptide, produced by multiple *Xenorhabdus* species, modulate insect immune responses and have potential as biocontrol agents in agriculture [18], further supporting the pharmaceutical relevance of this genus.

Comparative genomics of *Xenorhabdus* species have revealed significant variations in BGC content and organization, even among closely related strains, suggesting ecological adaptation and functional diversification [19,20,21]. However, despite the increasing interest in *Xenorhabdus* as a reservoir of bioactive compounds, much of its biosynthetic potential remains unexplored, particularly in newly described or geographically isolated strains. Here, we present the whole-genome sequencing and biosynthetic analysis of two *Xenorhabdus thailandensis* strains, ALN7.1 and ALN11.5, a novel species recently reported in 2024 [22], isolated from *Steinernema lamjungense* collected in Lamphun Province, Thailand. High-quality hybrid genome assemblies were generated for both strains, enabling comprehensive annotations and analyses. Using genome-based taxonomy, functional annotation, and secondary metabolite prediction, we examined their taxonomic placement, genomic characteristics, and biosynthetic potential to reveal novel metabolic pathways and assess their potential as sources of new natural products.

## 2. Materials and Methods

### 2.1. Bacterial Strains and Genome Sequencing

Bacterial strains previously isolated from *Steinernema lamjungense* eALN 7.1 and eALN 11.5, collected from agricultural areas in Lamphun Province in 2020, were designated bALN 7.1 and bALN 11.5, respectively [23]. In a previous study, *recA* gene sequence analysis of bALN 7.1 and bALN 11.5 exhibited less than 97% sequence similarity to *Xenorhabdus ehlersii* DSM 16337, suggesting that they may represent a distinct species [23]. Therefore, whole-genome sequencing was performed. Briefly, each bacterial strain was cultured by inoculating a single colony into Lysogeny Broth (LB; Sigma-Aldrich, Buchs, Switzerland) and incubated at 28 °C with shaking at 180 rpm for 24 h. DNA was extracted using the GenFind V2 kit (Beckman Coulter, Brea, CA, USA) according to the manufacturer’s protocol. For short-read sequencing, DNA libraries were prepared using the Illumina DNA Prep kit with quarter-volume reactions and sequenced on the Illumina NextSeq 500 platformto generate 150 bp paired-end reads (Illumina, Inc., San Diego, CA, USA). For long-read sequencing, libraries were prepared using the SQK-NBD114-96 and SQK-RBK114-96 kits (Oxford Nanopore Technologies, Oxford, UK) and sequenced on FLO-MIN106D (R9.4.1) flow cells using the GridION Mk1 platform. ONT base calling was performed using Guppy v6.0 [24]. All sequencing processes were performed at the Doherty Applied Microbial Genomics, The University of Melbourne, Melbourne, Australia.

### 2.2. Genome Assembly and Annotation

In preparation for assembly, raw Illumina and Nanopore sequencing reads were subjected to quality control using FastQC v0.12.1 [25]. Illumina reads were then quality-trimmed and filtered using Trimmomatic v0.39 [26], and parameters were applied to remove adapters, low-quality bases (Phred score < 20), and short reads (<36 bp). Nanopore reads were filtered using Filtlong v0.2.1 [27], retaining the top 90% of reads by quality and setting a minimum read length of 1000 bp to exclude short and low-quality reads. Hybrid genome assembly was performed using Unicycler v0.5.0 [28] in hybrid mode, integrating high-accuracy Illumina reads with long Nanopore reads to generate a high-contiguity assembly. Assembly quality was assessed using QUAST v5.2.0 [29], and metrics such as N50, total assembly length, and number of contigs were evaluated. For hybrid assembly polishing, Illumina short-read data were used to refine the draft assembly generated using Unicycler. First, Illumina paired-end reads were aligned to the assembly using BWA-MEM v0.7.17 [30]. The sorted BAM file was indexed using the samtools sort. Polishing was performed using Pilon v1.24 [31] with the command pilon --genome assembly.fasta --frags aln.sorted.bam --output pilon_polished --threads 8 --changes --vcf. This process corrected base-level errors by leveraging aligned Illumina reads. The assembled genomes were annotated using Prokka v1.14.6 [32] with the default parameters.

### 2.3. Bioinfomatics Analyses

To infer phylogenomic relationships within the genus *Xenorhabdus*, 34 reference genomes were retrieved from the NCBI GenBank database, with accession numbers listed in Table S1. *Photorhabdus luminescens* ATCC 29999 was included as an outgroup, and the strains bALN7.1 and bALN11.5 were used as query genomes. All genomes were analyzed using the Type Strain Genome Server (TYGS) (https://tygs.dsmz.de, accessed on 19 May 2025) [33] for whole-genome-based taxonomic classification and phylogenomic tree reconstruction. The resulting phylogenomic tree was midpoint-rooted and visualized using iTOL [34]. Digital DNA–DNA hybridization (dDDH) values were computed using the Genome-to-Genome Distance Calculator (GGDC) v4.0, and Average Nucleotide Identity (ANI) values were calculated using FastANI [35]. Gene function analysis was performed using EggNOG-mapper v5.0 (http://eggnog5.embl.de, accessed on 19 May 2025) [36]. To assess the biosynthetic potential of the genomes, secondary metabolite gene clusters were predicted using antiSMASH v8.0 [37] and the sequence differences of the clusters were compared using Clinker [38] (https://cagecat.bioinformatics.nl, accessed on 19 May 2025).

## 3. Results

The complete genomes of strains bALN 7.1 and bALN 11.5 were sequenced to accurately determine their taxonomic identities and investigate their potential for secondary metabolite production.

### 3.1. Bacterial Strain Identification

**Figure 1:**
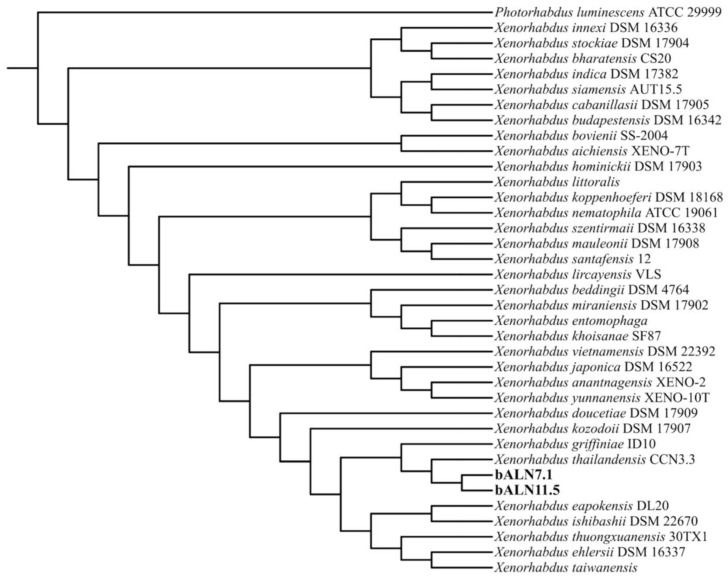
Whole-genome sequence-based phylogenomic tree constructed using the TYGS. The scale bar corresponds to 0.01 substitution per site. Bootstrap support values were calculated using 1000 replicates. The tree is midpoint-rooted.

First, to confirm their taxonomic placement, we performed a whole-genome analysis of strains bALN 7.1 and bALN 11.5 along with published genomes of related strains (Table S1) using the Type Strain Genome Server (TYGS). The resulting phylogenomic tree (Figure 1) revealed that both strains clustered within the same clade as *Xenorhabdus thailandensis* CCN3.3—a novel species described in 2024 [22]. In addition, average nucleotide identity (ANI) and digital DNA–DNA hybridization (dDDH) analyses were used to assess genomic similarity among bacterial strains based on whole-genome sequences. In this study, the ANI values between the strains bALN7.1 and bALN11.5 showed high similarity to the type strain *X. thailandensis* CCN3.3, with values of 98.37% and 98.30%, respectively. Correspondingly, the dDDH (formula d4) values were 87.2% and 87.1% (Table S2). These results strongly support the classification of bALN7.1 and bALN11.5 as *X. thailandensis*, in line with the current species thresholds of ANI ≥ 96% and dDDH ≥ 70% for species-level delineation [39,40,41]. Therefore, bALN7.1 and bALN11.5 were designated *X. thailandensis strains* ALN7.1 and ALN11.5, respectively.

### 3.2. Genome Characterization and Comparison

The genomes of *Xenorhabdus thailandensis* strains ALN7.1 and ALN11.5, sequenced and assembled in this study, exhibited high-quality assembly metrics and genomic features typical of the genus (Table 1). Both genomes were ~4.02 Mb in size, with 4,021,996 bp for ALN7.1 and 4,024,527 bp for ALN11.5. The number of coding sequences (CDSs) was 3448 and 3471, respectively, covering approximately 84.82% and 85.17% of their total genome sizes, which is consistent with the coding density observed in *Xenorhabdus* species [22,42,43]. In comparison, the reference strain *X. thailandensis* CCN3.3, which was isolated from a different province in Thailand, has a genome size of 3,794,703 bp with 3508 predicted proteins [22]. Despite these differences, the GC content remained consistent across all strains, with ALN7.1 and ALN11.5 at 43.33% and 43.36%, respectively, and CCN3.3 at 43.23%. Raw sequencing data for strains ALN7.1 and ALN11.5 were deposited in the NCBI Sequence Read Archive (SRA) under accession numbers SAMN48884708 and SAMN48884709, respectively.

In terms of assembly quality, the ALN strains were considerably more contiguous: ALN7.1 consisted of 49 contigs (N50:1,245,011 bp; L50:2), and ALN11.5 consisted of 19 contigs (N50:1,078,348 bp; L50:2). CCN3.3 was assembled into 133 scaffolds with an N50 of 199,653 bp and L50 of 9 [22]. These metrics help evaluate genome completeness and fragmentation, where an ideal assembly has a high N50 and low L50, indicating fewer but longer contigs [44]. This indicates that the majority of each ALN genome is contained within a small number of large contigs, which is further supported by high auN values (987,176 for ALN7.1 and 985,771 for ALN11.5). Structural elements such as rRNA operons (24 in ALN7.1 and 22 in ALN11.5), tRNAs (79 and 81, respectively), and the presence of a single tmRNA in both strains suggest nearly complete genome sequences. Notably, both strains also contained three annotated repeat regions, which are often associated with regulatory or mobile genetic elements.

### 3.3. Functional Genomics Analysis

**Figure 2:**
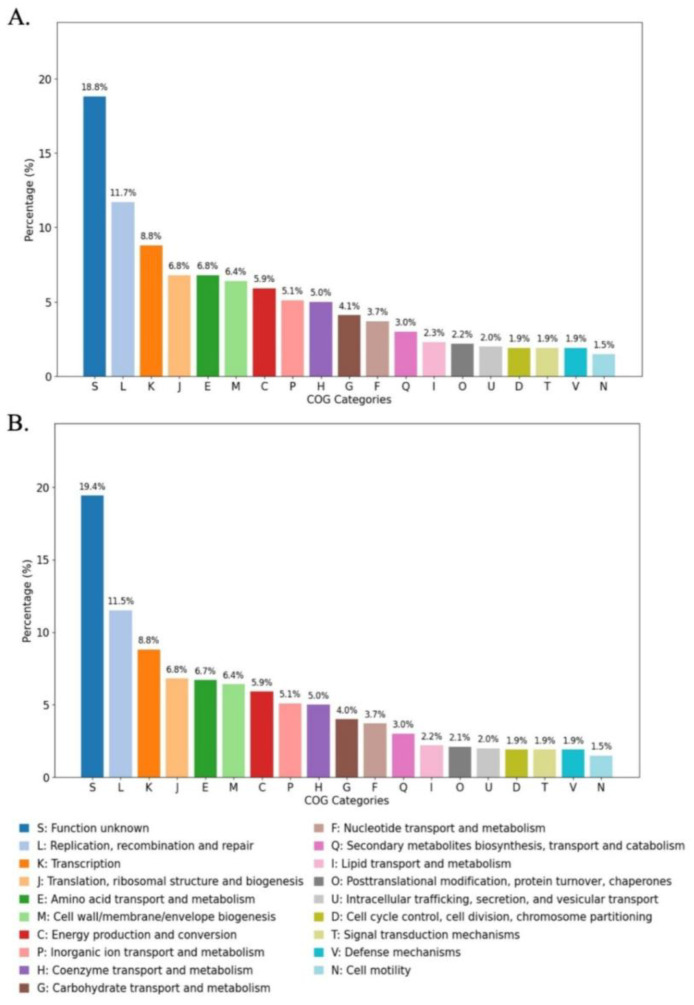
COG functional category distribution of predicted genes in *X. thailandensis* strains. (**A**) ALN 7.1 and (**B**) ALN 11.5. Percentages represent the proportion of genes assigned to each functional group.

The functional classification of genes in *X. thailandensis* strains ALN 7.1 and ALN 11.5 was performed using EggNOG-mapper v5.0 [36]. The resulting distribution of annotated genes into clusters of orthologous genes (COG) categories is shown in Figure 2. Both strains shared a similar COG profile, indicating conserved genomic features between the two isolates. The most abundant COG categories were typically those involved in translation, ribosomal structure and biogenesis, transcription, replication, recombination, repair, amino acid transport and metabolism, and carbohydrate transport and metabolism.

### 3.4. Elucidation of Biosynthetic Gene Clusters

The antiSMASH platform is a widely used tool for the identification and analysis of secondary metabolite biosynthetic gene clusters (BGCs) in bacterial and fungal genomes [37]. To predict the potential secondary metabolites produced by *X. thailandensis* strains ALN7.1 and ALN11.5, genome files in GenBank format were submitted to antiSMASH version 8.0 [37] for analysis. The results revealed 19 and 18 BGCs in strains ALN7.1 and ALN11.5, respectively (Table 2 and Table 3). These clusters spanned 867,040 bp in ALN7.1 and 851,810 bp in ALN11.5, accounting for approximately 21.56% and 21.16% of the total length of each genome, respectively, highlighting a substantial genomic investment in secondary metabolite production. Among the BGCs identified in strain ALN7.1, seven encoded nonribosomal peptide synthetases (NRPS), along with four hybrid clusters, one terpene, one azole-containing RiPP, one phenazine, and four classified as others. The BGC profile of strain ALN11.5 was largely similar, differing only by the presence of six NRPS clusters instead of seven; all other cluster types were identical between the two strains. In addition, strain ALN7.1 contains eight known BGCs alongside 11 uncharacterized clusters, whereas ALN11.5 possesses seven known and 11 novel clusters, highlighting its unexplored biosynthetic potential.

**Figure 3:**
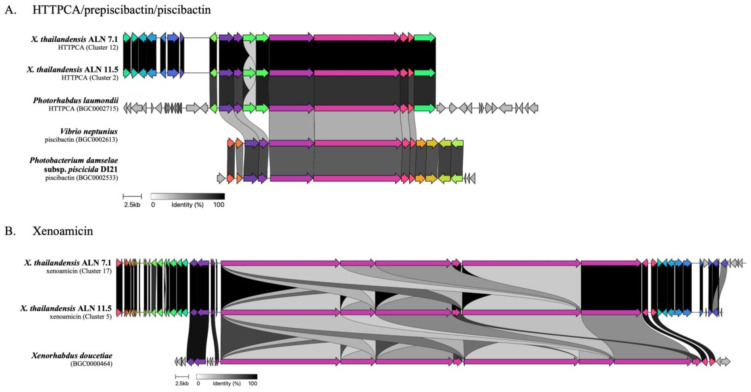
Clinker-generated gene cluster alignments of (**A**) HTTPCA/prepiscibactin/piscibactin and (**B**) xenoamicin. Connecting lines indicate homologous genes, with color intensity reflecting sequence similarity.

Further analysis revealed that the biosynthetic gene clusters (BGCs) for holomycin (absent in *X. thailandensis* ALN 11.5), pyrrolizixenamide A, hydrogen cyanide (HCN), bovienimide A, IOC, and gamexpeptide C in both *X. thailandensis* strains displayed high sequence identity to the known reference clusters. In contrast, the HTTPCA/prepiscibactin/piscibactin and xenoamicin A/B clusters exhibited moderate to low similarity based on alignments with public databases (Table 2 and Table 3). We then compared the sequence differences between the two clusters using Clinker [38] (https://cagecat.bioinformatics.nl, accessed on 19 May 2025). The results highlighted both conserved and divergent features of the HTTPCA/prepiscibactin/piscibactin BGCs among *Xenorhabdus* and other publicly available sequences from *Photorhabdus*, *Vibrio*, and *Photobacterium*. In *X. thailandensis* ALN 7.1 and ALN 11.5, the HTTPCA BGC (~42.7 kb) was nearly identical in terms of gene content, order, and orientation, with >90% nucleotide identity across most genes (Figure 3A). The preservation of NRPS-like core biosynthetic genes and associated transporter-encoding genes suggests a conserved biosynthetic logic, indicating evolutionary stability and potential selective pressure to retain this pathway. In contrast, the HTTPCA/prepiscibactin/piscibactin cluster in *Photorhabdus laumondii* (BGC0002715, ~56.5 kb) [45] shares only partial homology with *X. thailandensis* clusters. Core biosynthetic genes exhibited moderate identity (60–80%), whereas flanking and accessory genes were highly divergent or absent. Similarly, piscibactin-associated clusters from *Vibrio neptunius* (BGC0002613, ~32.2 kb) [46] and *Photobacterium damselae* subsp. *piscicida* DI21 (BGC0002533, ~35.2 kb) [47] shared structural similarity to the HTTPCA clusters, particularly in the central region encoding core biosynthetic and transporter genes. Up to 60% amino acid sequence identity is shared among the HTTPCA BGCs, suggesting that these clusters may be functional analogs or evolutionary derivatives of a common biosynthetic lineage. The conservation of core genes across the HTTPCA/piscibactin clusters points to a shared ancestral origin, followed by lineage-specific diversification through horizontal gene transfer and genomic rearrangement. Conserved regions likely encode essential biosynthetic functions, whereas variable segments contribute to the structural diversification and ecological adaptation of the resulting metabolites [48]. The xenoamicin BGCs in *X. thailandensis* strains ALN7.1 (~115.7 kb) and ALN11.5 (~120.1 kb) exhibited high sequence conservation with the characterized xenoamicin cluster in *X. doucetiae* (BGC0000464, ~105.9 kb) (Figure 3B). The core PKS-NRPS hybrid genes essential for xenoamicin biosynthesis were well conserved in terms of gene content, order, and orientation across all three strains, suggesting a shared biosynthetic framework. However, variations were observed in the flanking regions, including genes potentially involved in regulation, tailoring, or transport, particularly in *X. doucetiae*. This may influence the structure, regulation, and bioactivity of xenoamicin and its analogs.

### 3.5. Unknown BGCs in *Xenorhabdus thailandensis*

**Figure 4:**
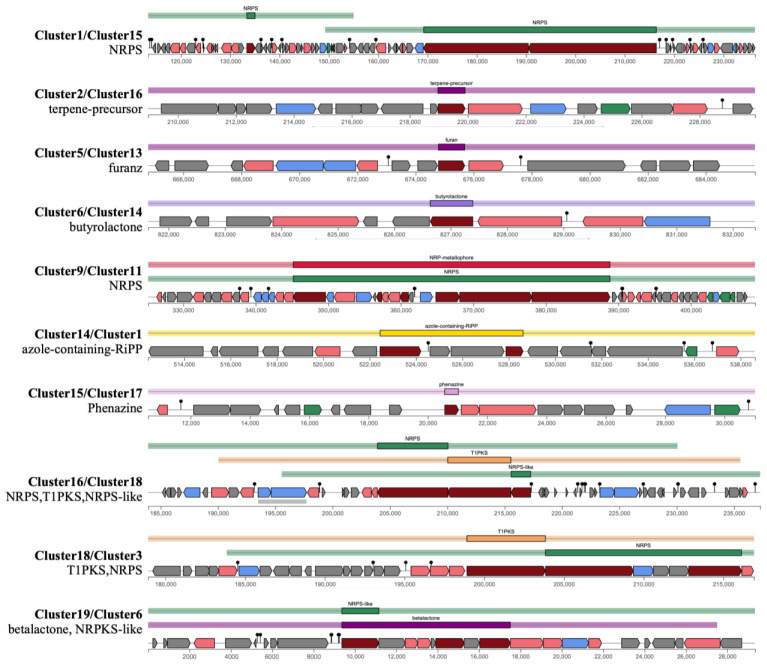
Uncharacterized biosynthetic gene clusters (BGCs) in *X. thailandensis* ALN 7.1 and ALN 11.5.

AntiSMASH analysis of *X. thailandensis* ALN 7.1 and ALN 11.5 revealed 11 uncharacterized biosynthetic gene clusters (BGCs) (Figure 4). Cluster 1 (ALN 7.1)/Cluster 15 (ALN 11.5) was annotated as a non-ribosomal peptide synthetase (NRPS) cluster and contained key genes involved in inositol catabolism (e.g., *iolG*, *iolE*, *iolB*), which are commonly associated with aminocyclitol biosynthesis. Inositol dehydrogenases have been identified in the biosynthetic pathways of aminoglycoside antibiotics, such as gentamicin and kanamycin, highlighting their relevance [49,50]. Genes involved in the degradation of aromatic compounds (e.g., 4-hydroxyphenylacetate 3-monooxygenase and 3,4-dihydroxyphenylacetate dioxygenase) were also present, suggesting metabolic versatility [51]. Several helix-turn-helix (HTH)-type transcriptional regulators (e.g., *HexR* and *FarR*) were detected, implying tightly regulated gene expression in response to environmental or cellular cues [52]. These findings suggest that these clusters may contribute to both primary and secondary metabolism, integrating complex carbon source utilization with natural product biosynthesis.

Cluster 2 (ALN 7.1)/Cluster 16 (ALN 11.5) appeared to be a terpene-precursor gene cluster, although no specific compound could be predicted. Terpene biosynthesis in bacteria remains relatively understudied, but it includes metabolites with antimicrobial, cytotoxic, and signaling functions [53,54].

Cluster 5 (ALN 7.1)/Cluster 13 (ALN 11.5) contained genes related to biosynthesis, transport, and RNA modification. The presence of key enzymes, such as zinc-type alcohol dehydrogenase and dihydrodiol dehydrogenasesuggests their roles in oxidative transformations [55], potentially linked to furan biosynthesis. Colistin resistance proteins (EmrA and EmrB) were also detected, implying mechanisms of self-protection against toxic or antimicrobial metabolites [56]. Additionally, multiple hypothetical proteins indicate unexplored functions within this region.

Cluster 6 (ALN 7.1)/Cluster 14 (ALN 11.5) included genes associated with regulation, glycerol metabolism, and transport. The presence of glycerol kinase and uptake proteins suggests the utilization of glycerol as a precursor for butyrolactone biosynthesis. Transporters and multidrug resistance proteins likely mediate the export and detoxification of metabolites. A rifampicin monooxygenase gene hinted at possible antibiotic modification, while enzymes from the methylerythritol phosphate (MEP) pathway may supply isoprenoid precursors for biosynthesis [57].

Cluster 9 (ALN 7.1)/Cluster 11 (ALN 11.5), annotated as an NRPS and NRP-metallophore hybrid cluster, included genes related to central metabolism, iron acquisition, and antibiotic biosynthesis. Enzymes such as acetate kinase and succinyl-related proteins suggest active carbon and nitrogen metabolism. Multiple siderophore biosynthesis and transport genes, including *enterobactin synthase* and *isochorismate synthase*, play a role in iron uptake. Large NRPS genes encode tyrocidine and gramicidin S synthases.

Cluster 13 (ALN 7.1)/Cluster 3 (ALN 11.5) featured genes encoding key biosynthetic enzymes such as linear gramicidin synthase subunits B and D. Additional genes involved in stress response (e.g., phage shock proteins), peptide and putrescine transport systems, and regulatory elements (e.g., *TyrR*, Psp operon activator) suggest that the cluster also supports adaptive responses to environmental pressures [58].

Cluster 14 (ALN 7.1)/Cluster 1 (ALN 11.5) contained genes predicted to encode azole-containing ribosomally synthesized and post-translationally modified peptides (RiPPs), a growing class of bioactive molecules with antimicrobial and antifungal activity [59]. Key biosynthetic components include *YcaO*, a methylthiotransferase essential for azole ring formation [60]. Additional genes were associated with amino acid metabolism (e.g., phosphoserine aminotransferase and serine–tRNA ligase), redox regulation (e.g., thioredoxin reductase), and transcriptional control (e.g., leucine-responsive regulatory protein). The presence of transport and replication-associated genes suggests the integration of biosynthetic activity with cellular physiology.

Clusters 15 (ALN 7.1)and 17 (ALN 11.5) encode key enzymes for phenazine biosynthesis, including *PhzB1*, *PhzD*, and *PhzG*. Phenazines are redox-active antibiotics with broad-spectrum activity, often associated with virulence in *Pseudomonas* species [61,62]. Their presence in *Xenorhabdus* may indicate a role in interspecies competition and oxidative stress defense.

Clusters 16 (ALN 7.1)and 18 (ALN 11.5) were annotated as hybrid NRPS–T1PKS–NRPS-like clusters, featuring core biosynthetic genes such as *surfactin synthase subunit 1*, a large multidomain NRPS-like protein, and *tyrocidine synthase 3*. Domain analysis revealed a modular architecture incorporating NRPS and PKS components (e.g., A, C, KS, AT, KR, and TD), indicative of a complex biosynthetic assembly line likely responsible for synthesizing a hybrid peptide-polyketide compound. Multiple transport-related genes (e.g., *Stp*, *MdtA*, *BepE*, *CvaA*, *and LtxB*) suggest mechanisms for compound export or self-resistance [63]. Tailoring enzymes, including methyltransferases and peptide deformylases, may perform post-synthetic modifications that enhance the bioactivity or stability of these peptides.

Clusters 18 (ALN 7.1)/3 (ALN 11.5) and 19 (ALN 7.1)/6 (ALN 11.5) were predicted to be hybrid T1PKS–NRPS and NRPS-like–betalactone clusters, respectively. Although less common, betalactone-containing BGCs have been implicated in the production of antifungal and antibacterial agents [64]. The co-occurrence of PKS, NRPS, and betalactone-associated genes may indicate the potential for the biosynthesis of structurally novel compounds.

## 4. Discussion

Whole-genome analyses confirmed that the bacterial isolates previously known as bALN7.1 and bALN11.5, recovered from *Steinernema lamjungense*, belonged to the recently described species *Xenorhabdus thailandensis*. This classification is strongly supported by high average nucleotide identity and digital DNA–DNA hybridization values that exceed the accepted thresholds for species delineation [39,40,41]. Confirmation of their taxonomic status resolves previous uncertainties based on single-gene analysis and establishes a basis for further comparative studies. Moreover, the genomic features of *X. thailandensis* strains ALN7.1 and ALN11.5 were generally consistent with those of the reference strain CCN3.3 [22], although both ALN strains had slightly larger genomes but fewer predicted coding sequences. This may be due to differences in annotation methods or strain-specific expansions of non-coding regions, which could reflect geographic variations within the species [21]. Overall, these findings indicate that strains ALN7.1 and ALN11.5 are closely related to *X. thailandensis* CCN3.3, yet exhibit improved assembly quality and minor differences in gene content. Further functional studies will provide valuable insights into the biological significance of these variants.

Therefore, we performed a functional classification of genes in *X. thailandensis* strains ALN 7.1 and ALN 11.5. The results revealed that the most abundant functional features were consistent with essential cellular functions and core metabolic processes. High representation in categories such as translation, transcription, and replication suggests robust mechanisms of gene expression and genome maintenance. Enrichment in metabolism-related categories, including amino acid and carbohydrate transport and metabolism, indicates the strains’ capacity for nutrient acquisition and environmental adaptability. These traits are typical of *Xenorhabdus* species, which engage in symbiotic and pathogenic interactions with nematodes and insect hosts [11,65].

Further analysis of BGCs in *X. thailandensis* strains ALN 7.1 and ALN11.5 revealed several clusters with high similarity to known compounds, such as holomycin, pyrrolizixenamide, hydrogen cyanide, bovienimide A, IOC, and gamexpeptide C. Holomycin is a dithiolopyrrolone-class antibiotic first identified in *Streptomyces* spp. It exhibits broad-spectrum antibacterial activity, especially against gram-positive bacteria, by inhibiting RNA synthesis [66,67,68]. Its absence in ALN 11.5 suggests possible strain-specific genome reduction, which may reflect ecological divergence or niche adaptation. The presence of the pyrrolizixenamide BGC, which is associated with bioactivity against eukaryotic cells, has been reported in other *Xenorhabdus* genomes and is thought to be involved in modulating host interactions or defense mechanisms [69,70]. For hydrogen cyanide (HCN), both *X. thailandensis* strains harbor a complete hydrogen cyanide synthase operon (*hcnABC*), indicating the genetic potential for HCN biosynthesis. To our knowledge, this is the first report of a putative HCN biosynthetic gene cluster in the *Xenorhabdus*-*Photorhabdus* clade. While HCN production is well documented in other entomopathogenic and plant-associated bacteria, such as *Pseudomonas aeruginosa* and *Burkholderia* spp. [71,72,73], its identification in *X. thailandensis* suggests a previously unrecognized metabolic capability. This may reflect an expanded repertoire of virulence factors or ecological strategies, potentially enhancing insecticidal activity or competitive fitness within the insect host or the soil environment. Both bovienimide A [74] and IOC [20] were identified in both strains, and BGCs are known to be restricted *to Xenorhabdus* and *Photorhabdus* species. Gamexpeptide C, a cyclized nonribosomal peptide first characterized by Nollmann et al. (2015) [18], demonstrates multifaceted role in bacterial physiology and interactions with insects, including immune modulation, virulence, and metabolic processes [75]. Collectively, the high sequence conservation of these BGCs in both *X. thailandensis* strains indicates that the key secondary metabolites involved in pathogenicity, competition, and symbiosis are evolutionarily conserved. The observed variation in the presence or absence of specific clusters (e.g., holomycin) may reflect strain-specific ecological specialization or differences in regulatory architecture.

Meanwhile, the HTTPCA/prepiscibactin/piscibactin and xenoamicin A/B clusters exhibited only moderate to low similarity compared to publicly available sequences. Comparative analysis revealed that, while the core biosynthetic genes within these clusters are highly conserved, the accessory regions vary considerably. This pattern suggests that these clusters have undergone evolutionary divergence, likely driven by horizontal gene transfer and local adaptation [48]. Such divergence in accessory regions has been previously implicated in shaping the chemical diversity of secondary metabolites across *Xenorhabdus* and *Photorhabdus* species [76,77]. Tailoring enzymes encoded in these regions can introduce modifications such as hydroxylation, glycosylation, and methylation, potentially leading to unique compound variants [78]. Together, these findings suggest that while the core biosynthetic capability is conserved, genomic plasticity in the surrounding BGC architecture could generate functional diversity among strains. Experimental validation, such as comparative metabolomics (e.g., LC-MS/MS) or heterologous expression, would be valuable for confirming the impact of these genetic differences on the production and structure. In addition, the diverse repertoire of unknown BGCs in both strains, including hybrid architectures and rare cluster types (e.g., betalactone, azole-RiPPs, and phenazines) [76,79,80], underscores their potential as a source of novel bioactive compounds. Future work should focus on metabolomic profiling to confirm compound production and structure elucidation using MS/MS and NMR techniques.

## 5. Conclusions

This study confirms that the entomopathogenic strains ALN7.1 and ALN11.5 belong to *Xenorhabdus thailandensis* based on high-quality genome assemblies. Both genomes exhibit characteristic features of symbiotic and pathogenic lifestyles and harbor diverse biosynthetic gene clusters (BGCs), including conserved pathways for known metabolites and uncharacterized clusters with the potential for novel compound production. Strain-specific differences in BGC profiles suggest ecological adaptation and functional divergence. These findings establish ALN7.1 and ALN11.5 as promising genomic resources for natural product discovery, with future work needed to explore their metabolomic output and biotechnological applications, especially for the discovery and development of novel antibiotics.

## Figures and Tables

**Table 1:** Genomic features of *X. thailandensis* strains assembled in the present study.

Statistics	*X. thailandensis* Strain ALN7.1	*X. thailandensis* Strain ALN11.5
Genome size (bp)	4,021,996	4,024,527
Number of contigs	49	19
Largest contig	1,262,586	1,303,736
CDS	3448	3471
rRNA	24	22
repeat_region	3	3
tRNA	79	81
tmRNA	1	1
N50	1,245,011	1,078,348
N90	216,926	406,632
auN	987,176	985,771
L50	2	2
L90	5	4
GC content (%)	43.33	43.36
NCBI accession number	SAMN48884708	SAMN48884709

**Table 2:** Prediction of biosynthetic gene clusters of secondary metabolites in *X. thailandensis* strain ALN 7.1 using antiSMASH 8.0.

Cluster	Type	Similarity Confidence	Most Similar Known Cluster	Gene Cluster from Organisms	MiBiG Accession/Reference
Cluster 1 Region 1.1	NRPS		unknown		
Cluster 2 Region 1.2	Terpene-precursor		unknown		
Cluster 3 Region 1.3	NRPS	High	holomycin	*Photobacterium galatheae*	BGC0002412
Cluster 4 Region 1.4	NRPS	High	pyrrolizixenamide A	*Xenorhabdus szentirmaii* DSM 16338	BGC0001873
Cluster 5 Region 1.5	Other: furanz		unknown		
Cluster 6 Region 1.6	Other: butyrolactone		unknown		
Cluster 7 Region 2.1	Other: hydrogen-cyanide	High	hydrogen cyanide	*Pseudomonas fluorescens*	BGC0002345
Cluster 8 Region 2.2	NRPS	High	bovienimide A	*Xenorhabdus bovienii* SS-2004	BGC0002135
Cluster 9 Region 2.3	NRPS		unknown		
Cluster 10 Region 2.4	Other: betalactone		IOC		
Cluster 11 Region 2.5	NRPS	High	gamexpeptide C	*Photorhabdus laumondii* subsp. *laumondii* TTO1	BGC0001128
Cluster 12 Region 3.1	Hybrid: NRPS, T1PKS	Low	HTTPCA/prepiscibactin/piscibactin	*Photorhabdus laumondii* subsp. *laumondii*	BGC0002715
Cluster 13 Region 3.1			unknown		
Cluster 14 Region 3.2	Azole-containing-RiPP		unknown		
Cluster 15 Region 4.1	Phenazine		unknown		
Cluster 16 Region 4.2	Hybrid: NRPS, T1PKS, NRPS-like		unknown		
Cluster 17 Region 5.1	NRPS	Medium	xenoamicin A/xenoamicin B	*Xenorhabdus doucetiae*	BGC0000464
Cluster 18 Region 5.2	Hybrid: T1PKS, NRPS		unknown		
Cluster 19 Region 8.1	Hybrid: betalactone, NRPKS-like		unknown		

**Table 3:** Prediction of biosynthetic gene clusters of secondary metabolites in *X. thailandensis* strain ALN 11.5 using antiSMASH 8.0.

Cluster	Type	Similarity Confidence	Most Similar Known Cluster	Gene Cluster from Organisms	MiBiG Accession/Reference
Cluster 1 Region 1.1	Azole-containing-RiPP		unknown		
Cluster 2 Region 1.2	Hybrid: NRP-metallophore, NRPS, T1PKS	Low	HTTPCA/prepiscibactin/piscibactin	*Photorhabdus laumondii* subsp. *laumondii*	BGC0002715
Cluster 3 Region 1.2			unknown		
Cluster 4 Region 1.3	Hybrid: NRPS, T1PKS		unknown		
Cluster 5 Region 1.4	NRPS	Medium	xenoamicin A/xenoamicin B	*Xenorhabdus doucetiae*	BGC0000464
Cluster 6 Region 1.5	Hybrid: betalactone, NRPKS-like		unknown		
Cluster 7 Region 1.6	Other: hydrogen-cyanide	High	hydrogen cyanide	*Pseudomonas fluorescens*	BGC0002345
Cluster 8 Region 1.7	NRPS	High	bovienimide A	*Xenorhabdus bovienii* SS-2004	BGC0002135
Cluster 9 Region 2.1	NRPS	High	gamexpeptide C	*Photorhabdus laumondii* subsp. *laumondii* TTO1	BGC0001128
Cluster 10 Region 2.2	Other: betalactone		IOC		
Cluster 11 Region 2.3	NRPS		unknown		
Cluster 12 Region 3.1	NRPS	High	pyrrolizixenamide A	*Xenorhabdus szentirmaii* DSM 16338	BGC0001873
Cluster 13 Region 3.2	Other: furan		unknown		
Cluster 14 Region 3.3	Other: butyrolactone		unknown		
Cluster 15 Region 4.1	NRPS		unknown		
Cluster 16 Region 4.2	Terpene-precursor		unknown		
Cluster 17 Region 5.1	Phenazine		unknown		
Cluster 18 Region 5.2	Hybrid: NRPS, T1PKS, NRPS-like		unknown		

## Data Availability

All relevant data are available in the article and its Supporting Information Files.

## Supplementary Materials

The following supporting information can be downloaded at: https://www.mdpi.com/article/10.3390/biology14080905/s1, Table S1: Reference genomes were obtained from NCBI genome database; Table S2: Pairwise comparisons of *Xenorhabdus* spp. genomes.

## Abbreviations

The following abbreviations are used in this manuscript:

BGCs	Biosynthetic gene clusters
TYGS	The Type Strain Genome Server
dDDH	DNA–DNA hybridization
GGDC	Genome-to-Genome Distance Calculator
ANI	Average Nucleotide Identity
COG	Clusters of orthologous genes
